# Adaptive reinforcement learning for lithography optimization: a scalable AI-driven solution for next-generation semiconductor manufacturing

**DOI:** 10.1038/s41598-026-43555-z

**Published:** 2026-03-15

**Authors:** Umar Rashid, Fahad Shafique, Hamza Atif, Waleed Waheed, Rizwan Khan, Muhammad Akmal

**Affiliations:** 1 Department of Electrical Engineering, University of Engineering and Technology, New Campus, Lahore, Pakistan; 2 Department of Electrical, Electronic, and Future Technologies, Sheffield Hallam University, Sheffield, S1 1WB UK; 3 R&D Consultant , Appifest, Sydney , Australia

**Keywords:** Computational lithography, Deep neural networks, Inverse lithography technology, Mask optimization, Reinforcement learning, Semiconductor manufacturing, Engineering, Materials science, Mathematics and computing, Nanoscience and technology, Optics and photonics

## Abstract

Semiconductor lithography, a pivotal process in integrated circuit (IC) fabrication, accounts for approximately 30% of production costs and faces significant challenges as feature sizes shrink to sub-nanometer scales. Optical diffraction and process-induced distortions complicate precise patterning, necessitating advanced techniques beyond traditional Optical Proximity Correction (OPC). Inverse Lithography Technology (ILT) offers a mathematically robust approach to enhance pattern fidelity, yet its high computational complexity limits scalability. We propose Adaptive Reinforcement Learning for Lithography Optimization (ARLO), a U-Net-based framework integrating self-attention mechanisms and reinforcement learning (RL) to iteratively optimize photomasks using real-time lithographic simulations. Evaluated on the LithoBench benchmark, ARLO achieves a 37.8% reduction in $L_2$ Loss and a 74.0% reduction in Process Variation Band (PVB) compared to GAN-OPC, alongside 14.7% and 9.1% $L_2$ Loss reductions and 51.3% and 37.1% PVB reductions versus Deep LithoNet (DLN) and RL-ILT, respectively. Despite a higher shot count (181.4% increase vs. GAN-OPC, 59.0% vs. DLN-1, 29.4% vs. RL-ILT), ARLO maintains a competitive runtime of 0.035 seconds per patch. These results position ARLO as a scalable, efficient solution for next-generation semiconductor manufacturing.

## Introduction

Semiconductor lithography serves as the foundation of modern integrated circuit (IC) fabrication, enabling the precise delineation of nanoscale patterns onto silicon wafers, a process critical to the realization of advanced microelectronic devices. As Moore’s Law propels the industry toward feature sizes below 3 nm, lithography faces unprecedented challenges stemming from optical diffraction effects, process-induced variations, and the fundamental resolution limits of optical systems^1,2^. These obstacles not only complicate the accurate transfer of patterns from mask to wafer but also significantly inflate production costs. Notably, lithography accounts for approximately 30% of total IC fabrication expenses, underscoring the urgent need for innovative techniques to maintain pattern fidelity and manufacturability at cutting-edge technology nodes^3^.

Historically, Optical Proximity Correction (OPC) has been the cornerstone of computational lithography, pre-adjusting mask designs to mitigate distortions arising from optical and process effects^4,5^. This technique has evolved considerably, yet its efficacy diminishes as feature sizes approach the wavelength of extreme ultraviolet (EUV) light (13.5 nm), where scalability issues and difficulties in capturing global pattern dependencies become pronounced^6,7^. In response, Inverse Lithography Technology (ILT) has emerged as a mathematically rigorous alternative, reformulating mask synthesis as an inverse problem to optimize photomasks for enhanced wafer print accuracy^8^. Despite its potential, ILT’s practical deployment in high-volume manufacturing (HVM) is curtailed by its dependence on iterative, computationally demanding solvers and its propensity to generate complex mask geometries that challenge manufacturability^9,10^.

To address these limitations, we propose Adaptive Reinforcement Learning for Lithography Optimization (ARLO), a pioneering framework that integrates deep neural networks (DNNs) and reinforcement learning (RL) to revolutionize mask optimization. ARLO employs a U-Net architecture augmented with self-attention mechanisms to deliver rapid initial mask predictions, followed by an RL agent that iteratively refines these masks based on real-time lithographic simulation feedback. Evaluated on the LithoBench benchmark, ARLO achieves a 37.8% reduction in $L_2$ Loss and a 74.0% reduction in Process Variation Band (PVB) compared to GAN-OPC^9^, alongside 14.7% and 9.1% $L_2$ Loss reductions and 51.3% and 37.1% PVB reductions versus Deep LithoNet (DLN)^11^ and RL-ILT^12^, respectively. Despite a higher shot count (181.4% increase vs. GAN-OPC, 59.0% vs. DLN-1, 29.4% vs. RL-ILT), ARLO maintains a competitive runtime of 0.035 seconds per patch. This hybrid approach, shown in Fig. 3, integrates the efficiency of DNN-based inference with the flexibility of RL-driven optimization to provide a scalable solution for advanced lithography processes.

In particular, RL-OPC proposed in^13^, which uses deep RL for pixel-level mask adjustments, achieving $\approx 15\%$ EPE reduction on 45nm nodes but with higher convergence steps (avg. 25) than our ARLO (12 steps). Furthermore, recent RL extensions like CAMO^14^ and MLP based GMUNet-ILT^15^ address mask complexity but overlook real-time simulation feedback, which ARLO uniquely integrates.

### Motivation and challenges

The motivation for developing ARLO arises from the escalating complexity of sub-5-nm technology nodes, where conventional lithography techniques falter under the dual imperatives of precision and production scalability. As transistor dimensions shrink, the interplay of optical diffraction, process variability, and equipment limitations imposes stringent requirements on mask design and optimization. Traditional OPC and ILT methods, while effective in earlier nodes, struggle to meet these demands due to several critical challenges:

- Computational overhead: iterative ILT solvers, reliant on gradient-based optimization, incur substantial runtime penalties, rendering them impractical for full-chip mask synthesis within the time constraints of modern manufacturing workflows^9^. This computational burden limits their applicability in real-time production environments, where rapid turnaround is essential.
- Mask complexity: ILT-generated masks often exhibit highly fragmented and irregular features, significantly increasing the shot count in electron-beam lithography processes. This complexity not only elevates manufacturing costs but also poses challenges for mask inspection and repair, hindering adoption in HVM settings^16^.
- Process robustness: variability in focus, dose, and other process parameters necessitates masks that maintain pattern fidelity across a wide range of conditions. Existing methods frequently lack the adaptability to ensure consistent performance under such variability, compromising yield and reliability^17^.

These challenges collectively underscore the need for a transformative approach capable of balancing computational efficiency, mask simplicity, and robustness, a need that ARLO seeks to fulfill through its innovative integration of AI-driven techniques.

### Contributions

This paper presents a comprehensive advancement in computational lithography through the introduction of the ARLO framework, accompanied by rigorous evaluation and practical validation. The key contributions of this work are as follows:

- ARLO framework: we introduce a U-Net-based ILT solution that leverages reinforcement learning for real-time mask refinement. By combining rapid DNN inference with RL-driven optimization, ARLO achieves unparalleled improvements in printability and manufacturability, addressing the shortcomings of traditional ILT and OPC methods. This framework dynamically adapts to lithographic feedback, ensuring masks are optimized for both accuracy and production feasibility.
- LithoBench evaluation: through extensive benchmarking using the LithoBench platform, we provide a detailed comparison of ARLO against state-of-the-art approaches, including GAN-OPC^9^, CFNO^9^, Deep LithoNet (DLN)^11^, and RL-ILT^12^. This evaluation demonstrates significant enhancements across critical metrics, including runtime efficiency, mask fidelity, and process variation tolerance, establishing ARLO as a leading solution in the field.
- Scalability demonstration: we validate ARLO’s capability to perform real-time mask optimization with reduced computational overhead, making it a viable candidate for production-scale deployment. This scalability bridges the gap between academic research and industrial application, offering a practical pathway for integrating advanced AI techniques into semiconductor manufacturing workflows.

By addressing the core limitations of existing lithography methods, ARLO positions itself as a transformative tool for next-generation IC fabrication, with the potential to enhance yield, reduce costs, and accelerate the development of sub-nm technologies.

## Literature review

Traditional OPC methods, progressing from rule-based^4^ to model-based approaches^5^, have significantly improved lithographic accuracy. However, as semiconductor technology advances beyond 5 nm, diffraction effects, process variations, and limitations of extreme ultraviolet (EUV) lithography render OPC inadequate for high-fidelity pattern transfer^6,7^. ILT redefines mask optimization as an inverse problem, computing photomask patterns that enhance edge placement accuracy and manufacturability^8,17^. Nevertheless, its iterative nature imposes substantial computational overhead and results in mask complexity, restricting scalability in high-volume production^9,10^.

Recent research has pivoted toward deep learning-based ILT solutions to accelerate mask optimization while maintaining printability. Deep Neural Networks (DNNs), particularly convolutional neural networks (CNNs), have been utilized to predict optimal mask corrections, reducing computational demands compared to traditional ILT frameworks^7^. Neural-ILT, introduced by Jiang et al., employs CNNs trained on lithography datasets to produce high-accuracy photomasks with reduced runtime^7^. Similarly, GAN-based methods such as GAN-OPC, proposed by Yang et al., leverage Generative Adversarial Networks (GANs) to refine mask optimization and minimize shot count, thereby improving manufacturability^17^. Jia and Lam pioneered stochastic gradient descent-based ILT optimization, highlighting the potential of AI-driven computational lithography^18^. Xu et al. introduced Deep LithoNet (DLN), a U-Net-based model with enhanced feature extraction for EUV lithography, achieving improved fidelity over GAN-OPC^11^. Kim et al. proposed RL-ILT, integrating reinforcement learning with ILT to dynamically adjust masks, offering robustness to process variations^12^.

**Fig. 1:**
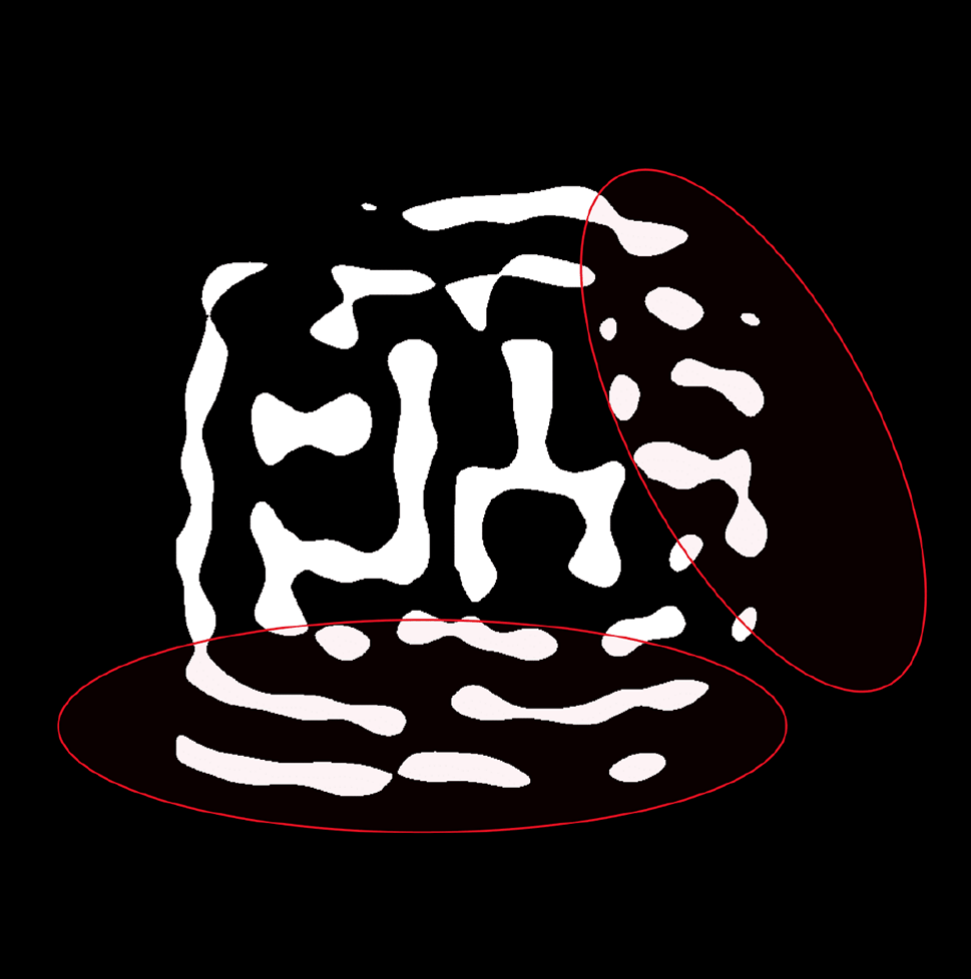
Metal layer tile from the LithoBench dataset, with the red highlighted region indicating Sub-Resolution Assist Features (SRAFs) designed to counteract diffraction effects and enhance print fidelity^10^.

Mask complexity remains a critical challenge in ILT, with traditional methods often generating intricate features that increase shot count and manufacturing costs. In sub-wavelength lithography, where the wavelength of light (e.g., 193 nm for DUV or 13.5 nm for EUV) exceeds pattern dimensions, diffraction scatters light, distorting the target image on the wafer. Adaptive Sub-Resolution Assist Features (SRAFs) have been developed to counteract this by introducing non-printing patterns to the mask, negating diffraction effects and ensuring accurate pattern transfer^10^. Figure 1 illustrates a metal layer tile from the LithoBench dataset, where the red highlighted region represents SRAFs strategically placed to mitigate diffraction effects, thereby improving pattern fidelity on the wafer. This approach, detailed in^10^, ensures accurate pattern transfer without printing the assist features themselves, a critical technique for sub-5-nm lithography.

Reinforcement Learning (RL)-based optimization marks a significant advancement, with AI agents learning optimal mask transformations through iterative lithography simulation feedback^19^. Adaptive Reinforcement Learning for Lithography Optimization (ARLO) employs RL to balance printability, mask complexity, and computational efficiency. Li et al. demonstrated that RL-assisted ILT reduces shot count and enhances printability, particularly for EUV lithography^20^. Rao et al. and Chen et al. extended deep RL to mask decomposition, reducing computation time while preserving manufacturability^21,22^. These RL-driven approaches adapt dynamically, providing a promising avenue for real-time mask correction^23^.

Physics-aware deep learning frameworks enhance ILT accuracy by incorporating real-world constraints into neural networks. Shi et al. introduced a feature vector physics-based approach, improving generalization across process conditions^24^. Ma et al.’s fast inverse lithography model, driven by dual-channel deep learning, integrates physical constraints for robust mask synthesis^25^. These advancements ensure that AI-driven ILT remains efficient and resilient to fabrication challenges^26,27^.

**Fig. 2:**
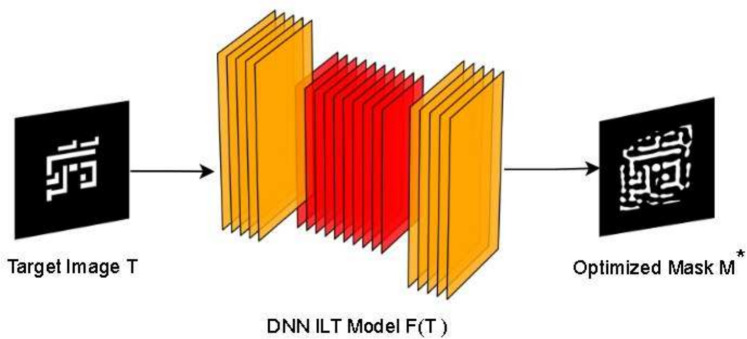
DNN-based ILT workflow: Target layout $T$ is transformed into an optimized mask $M$ using ARLO’s hybrid deep learning.

Hybrid ILT methodologies combining heuristic optimization with AI enhancements have also surfaced. Cecil et al.’s graph convolutional ILT model represents patterns as graph structures for efficient optimization^28^, while Zhang et al.’s Hybrid ILT-GAN fuses GANs with traditional solvers to accelerate mask correction^29^. Chen et al.’s block stacking CNN approach further optimizes computational efficiency for complex layouts^30^. These hybrid models exhibit strong potential for balancing speed and manufacturability^31,32^. Figure 2 depicts the DNN-based ILT workflow employed by ARLO, where a target layout $T$ is transformed into an optimized mask $M$ through a hybrid approach combining deep learning and reinforcement learning. This workflow, inspired by^7^, integrates initial mask prediction with iterative refinement to ensure high fidelity and manufacturability, addressing the computational inefficiencies of traditional ILT methods.

Benchmarking platforms such as LithoBench and OpenILT provide standardized datasets for evaluating ILT performance under varied conditions^9,33^. LithoBench, in particular, facilitates rigorous comparisons across synthetic and real-world IC layouts^9^. Lin et al.’s UNeXt-ILT framework leverages global context awareness, integrating deep learning with classical ILT to enhance efficiency and printability^32^. Zheng et al. emphasized the need for real-time ILT algorithms adaptable to multi-layer designs, a challenge RL-based models like ARLO are well-equipped to address^34,35^.

As the industry targets sub-nm nodes, integrating ARLO and hybrid AI-driven ILT methodologies will redefine computational lithography. These frameworks optimize efficiency and process window robustness, promising higher yields and reduced mask costs in high-volume production^18,28^. Emerging techniques, such as model-driven block stacking ILT and adversarial training-based optimization, continue to push scalability boundaries^30,36^. The evolution of ILT, propelled by AI and computational advancements, will be instrumental in addressing semiconductor manufacturing complexity, enabling efficient, high-precision, and scalable photomask solutions^37,38^.

## Related work

This section explores foundational advancements in lithography simulation and the transformative role of Inverse Lithography Technology (ILT) in mask optimization, providing the context for AI-driven enhancements in computational lithography.

### Lithography simulation in semiconductor manufacturing

As feature sizes approach atomic scales, often below 3 nm in advanced technology nodes, the physical constraints of photolithography, including diffraction limits and process-induced variations, become increasingly prominent^1,2^. The process is typically divided into two interdependent stages: optical projection, which models the interaction of light with the photomask, and photoresist modeling, which simulates the formation of the final resist pattern on the wafer.

The optical projection stage simulates light propagation through the lithography system to form an aerial image on the wafer surface, based on Hopkins’ diffraction theory^1^. The aerial image intensity $I(x, y)$ is modeled as:

1 $$\begin{aligned} I(x, y) = H(M) = \sum _{k=1}^{K} \mu _k \left| h_k \otimes M \right| ^2 \end{aligned}$$

where $h_k$ represents the $k$th optical kernel function, embodying the point spread function of the optical system, $\mu _k$ is its corresponding weight, $\otimes$ denotes convolution, and $M$ is the mask pattern^1^. The squared modulus $| \cdot |^2$ captures the intensity of diffracted light, accounting for interference effects under specific illumination conditions, such as partially coherent light^3,39^. For extreme ultraviolet (EUV) lithography, with its 13.5-nm wavelength, the model incorporates additional factors like flare and mask shadowing to ensure precision, reflecting the unique challenges of EUV systems.

The photoresist modeling stage translates the aerial image into the final printed resist image $Z(x, y)$, defining the physical circuit pattern on the wafer. This transformation is commonly approximated using a nonlinear sigmoid function to replicate the photoresist’s thresholding behavior^2^:

2 $$\begin{aligned} Z(x, y) = \sigma _Z(I(x, y)) = \frac{1}{1 + e^{-\alpha (I(x, y) - I_{th})}} \end{aligned}$$

In this equation, $I_{th}$ is the intensity threshold distinguishing exposed from unexposed regions, $\alpha$ governs the transition steepness (reflecting resist sensitivity), and $(x, y)$ denotes spatial coordinates^2^. This model simplifies the complex chemical and physical interactions within the photoresist, such as exposure, development, and diffusion, into a computationally manageable form^9^.

### Inverse lithography technology

ILT adopts a global approach, formulating mask synthesis as an inverse problem with the objective of determining a mask pattern $M(x, y)$ that, when processed through the lithography system, produces a resist image $Z(x, y)$ closely matching the target circuit layout $T(x, y)$^8^. This is achieved through a parameterized mask function, typically modeled with a sigmoid:

3 $$\begin{aligned} M(x, y) = \sigma _M(P(x, y)) = \frac{1}{1 + e^{-\beta (P(x, y) - \gamma )}} \end{aligned}$$

where $\beta$ controls the transition steepness, $\gamma$ is an offset, and $P(x, y)$ represents an intermediate optimization variable, such as a pixelated mask field^8^. The complete transformation from mask to wafer pattern is expressed as:

4 $$\begin{aligned} Z = \sigma _Z(H(\sigma _M(P))) \end{aligned}$$

The optimization process centers on minimizing a composite loss function $L_f$, which balances fidelity, robustness, and manufacturability^9^. Gradient descent and stochastic optimization techniques iteratively adjust $P(x, y)$ to minimize $L_f$, producing masks with superior fidelity and process tolerance^6,7^. However, this iterative approach incurs significant computational overhead, particularly for full-chip designs, prompting research into faster solvers and approximations, such as GPU-accelerated ILT, to enhance scalability and practicality in high-volume manufacturing settings.

## Adaptive reinforcement learning for lithography optimization (ARLO)

The Adaptive Reinforcement Learning for Lithography Optimization (ARLO) framework represents a significant advancement in photomask design for semiconductor manufacturing. This approach integrates a U-Net-based deep neural network with a reinforcement learning (RL) policy agent to iteratively refine masks, addressing critical limitations in traditional Inverse Lithography Technology (ILT). The primary objective of ARLO is to deliver masks that excel in printability, robustness to process variations, and manufacturability, while mitigating the computational overhead and complexity that hinder conventional ILT methods. Standard ILT techniques often rely on iterative gradient-based optimization or heuristic adjustments, which demand substantial computational resources and frequently produce masks with intricate geometries impractical for large-scale production. ARLO overcomes these challenges by leveraging deep learning for rapid initial mask generation and RL for precise, adaptive refinement, embedding real-world fabrication constraints, such as mask complexity and process variability, directly into the optimization process. The ARLO framework operates through a cyclical optimization loop, beginning with an initial mask prediction, evaluating its performance via lithography simulation, and refining it iteratively based on feedback, guided by an RL agent.

### Architectural Overview of ARLO

ARLO’s architecture comprises three core components designed to function synergistically:

- U-Net-based neural network: generates an initial mask prediction from a target circuit layout with high efficiency and spatial fidelity.
- Lithography simulation: assesses the mask’s performance by simulating its print on a silicon wafer under various conditions.
- Reinforcement learning agent: refines the mask iteratively, optimizing it based on simulation-derived performance metrics.

These elements form a cohesive system that evolves masks through successive iterations, ensuring alignment with both design intent and fabrication realities. Table 1 lists the key simulation and model parameters governing ARLO’s U-Net architecture, optimization process, and PyLitho simulation settings.

**Table 1:** Simulation and model parameters for the ARLO framework.

Parameter	Value
Kernel size	3x3
Padding	1
Stride	1
Pooling	MaxPoo$L_2$d(2, 2)
Upsampling	Bilinear (scale factor 2)
Activation	ReLU + sigmoid (output)
Optimizer	AdamW
Reward function	$-(L_2 + \text {PVB})$
KernelNum	24
TargetDensity	0.225
PrintThresh	0.5
PrintSteepness	100.0
DoseMax	1.02
DoseMin	0.98
DoseNom	1.00

#### U-Net for initial mask prediction

The U-Net architecture serves as the foundation for ARLO’s mask generation, utilizing its proven encoder-decoder structure to produce an initial mask tailored to the target layout. This component is critical for establishing a robust starting point, preserving essential spatial features while minimizing extraneous details that could complicate downstream optimization. The U-Net is structured as follows:

- Encoder (feature extraction): employs a sequence of convolutional layers, each followed by batch normalization and ReLU activation, to extract features from the input layout. Attention mechanisms are incorporated into each layer to focus on key lithographic features, such as dense patterns or sharp edges, and to capture long-range spatial relationships. Max-pooling operations progressively reduce spatial dimensions, condensing the data into a compact, abstract representation suitable for deeper analysis.
- Bottleneck (residual): consolidates extracted features into a high-dimensional space using multi-head attention layers, inspired by transformer architectures. This allows the model to evaluate global layout relationships by addressing manufacturing variations like focus shifts or dose changes.
- Decoder (mask reconstruction): reconstructs the mask by upsampling bottleneck features using bilinear interpolation. Skip connections from the encoder preserve high-resolution details, maintaining spatial accuracy throughout reconstruction. The output layer generates ten binary masks, each corresponding to a distinct lithography exposure condition (e.g., nominal, maximum, and minimum process corners), providing a basis for RL optimization.

**Fig. 3:**
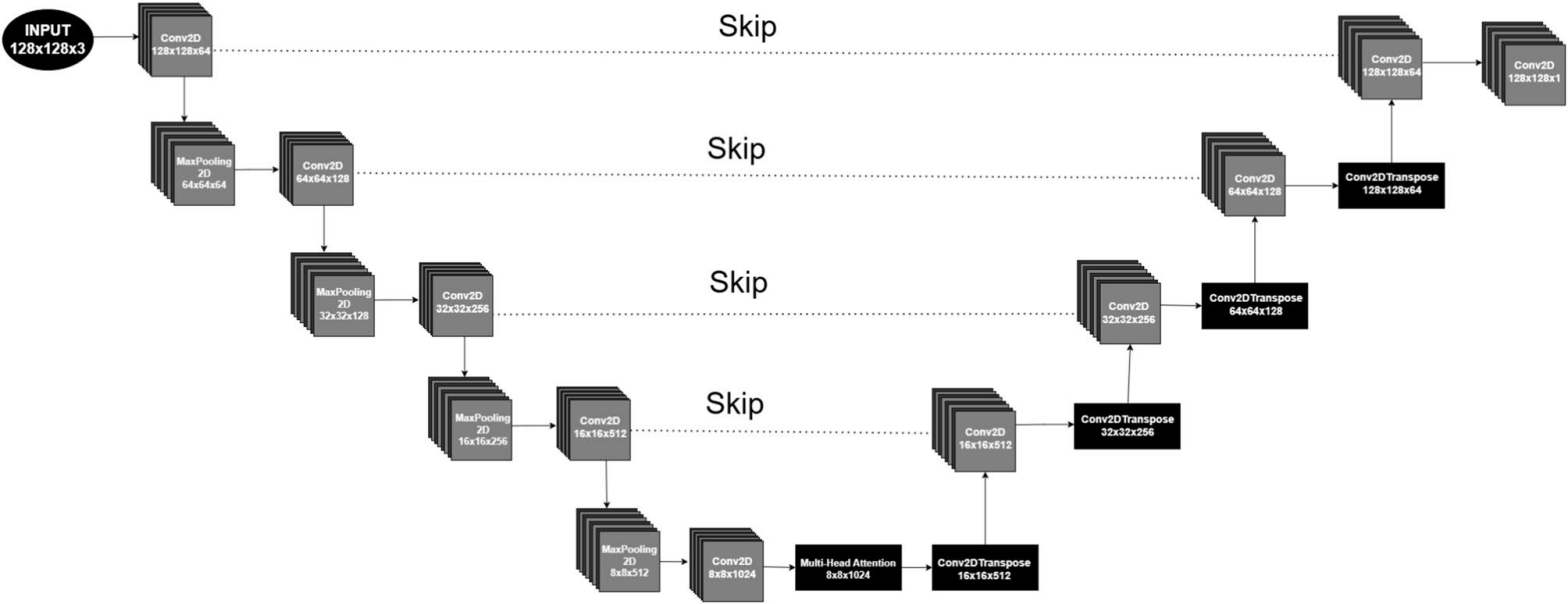
ARLO’s U-Net architecture for initial mask prediction, featuring an encoder–decoder structure with skip connections, convolutional layers (Conv2D), max-pooling (MaxPooling2D), and transposed convolutions (Conv2DTranspose) for upsampling^7^.

### Reinforcement learning for mask optimization

#### Role of the RL agent

The reinforcement learning agent is the cornerstone of ARLO’s optimization strategy, transforming the initial U-Net prediction into a finely tuned mask through an active refinement process. Unlike traditional deep learning models that produce a single, static output, the RL agent adjusts the mask based on real-time feedback from lithography simulations, optimizing its performance across complex trade-offs—between print fidelity, process stability, and mask simplicity—that static methods often fail to resolve.

The reinforcement learning (RL) agent operates on a continuous grayscale mask representation, where each pixel value corresponds to mask transmittance. The RL agent operates within a Markov Decision Process (MDP) framework, defined as:

- State ($s_t$): the state $s_t$ is defined as the current grayscale mask $M_t\in [0,1]^{N\times N}$.
- Action ($a_t$): at each iteration, the RL agent outputs a continuous action:
  $$\begin{aligned} a_t = \Delta M_t \end{aligned}$$
  representing bounded pixel-level transmittance updates, constrained as:
  $$\begin{aligned} \Delta M_t \in [-\delta , \delta ] \quad \delta = 0.1 \end{aligned}$$
  Thus, the updated mask is computed as:
  $$\begin{aligned} M_{t+1} = \text {clip}(M_t + \Delta M_t, 0.1) \end{aligned}$$
  Binary mask geometry is obtained only after optimization, via thresholding during evaluation. The RL agent does not explicitly move geometric edges or boundary segments.This formulation aligns ARLO with pixel-level ILT methods while enabling smooth optimization dynamics and stable PPO training.
- Reward ($r_t$): a quantitative measure of the mask’s performance, derived from simulation results. The agent receives a scalar reward $r_t$ at each iteration to guide mask refinement:
  5 $$\begin{aligned} r_t = -w_1L_2 - w_2 PVB -w_3EPE + w_4\cdot \frac{1}{shot\_count + \epsilon } \end{aligned}$$
  Each term reflects a critical lithography objective, and the weights $\{w_1, w_2, w_3, w_4\}$ were systematically tuned to balance competing goals.
- Policy ($\pi$): the agent’s learned strategy for selecting actions to maximize cumulative rewards.

**Fig. 4:**
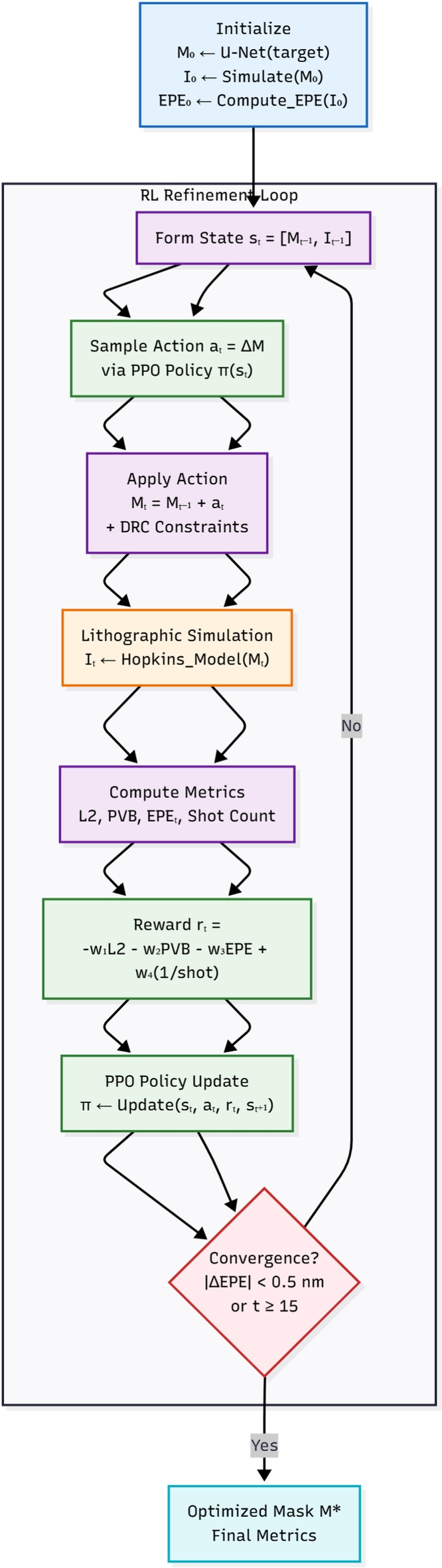
ARLO reinforcement learning pipeline. The U-Net generates an initial mask, followed by a PPO-driven refinement loop (10–15 steps) with real-time lithographic feedback. Convergence is reached when $|\Delta \text {EPE}| < 0.5$ nm.

ARLO employs the Proximal Policy Optimization (PPO) algorithm to ensure stable and efficient learning, balancing exploration and exploitation while mitigating instability. Figure 4 that visualizes 5 iterations via pattern (e.g., Iteration 1: +2nm edge shift resolves hotspot; Iteration 5: Final mask with 92% fidelity). This clarifies agent behavior and simulation loop (Hopkins-based feedback every step).

#### Lithography simulation and reward computation

The lithography simulation environment evaluates each mask by modeling its optical projection and resist development, producing a simulated wafer print under nominal and extreme conditions. The evaluation is guided by four key metrics:

- $L_2$ loss (printability error)^9^:
  6 $$\begin{aligned} L_2 = \Vert Z_{nom} - T \Vert _2^2 \end{aligned}$$
- Process variation band (PVB)^9^:
  7 $$\begin{aligned} \text {PVB} = \Vert Z_{max} - Z_{min} \Vert _2^2 \end{aligned}$$
- Edge placement error (EPE)^7^:
  8 $$\begin{aligned} EPE = \sum _{i=1}^{N} (x_i - x_{target})^2 \end{aligned}$$
- Shot count (mask complexity)^9^:
  9 $$\begin{aligned} Shots = \sum _{i=1}^{N} n1(M_i > 0.5) \end{aligned}$$

Here L2 penalizes intensity mismatch via MSE on aerial images while PVB achieves this via min-max contour variance. The reward function is computed using (5)^7^ where $w_1, w_2, w_3, w_4$ are weights calibrated to balance objectives^7^.

**Algorithm 1:**
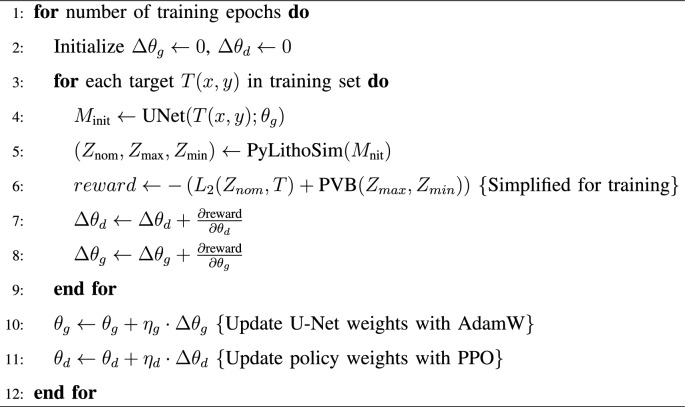
ARLO RL training. Input: target layout image *T*(*x*, *y*). Output: optimized mask $M_{\text {opt}}$ and updated weights $\theta _g, \theta _d$.

### Model training and optimization

subsubsectionTraining process

Training ARLO involves a two-phase strategy. The U-Net is pretrained on a dataset of target-mask pairs using binary cross-entropy loss, followed by joint training with the RL agent adapting to simulation feedback. Key aspects include:

- Optimization: uses the AdamW optimizer with a cosine annealing learning rate schedule (from $10^{-3}$ to $10^{-6}$).
- Computational efficiency: processes 256 masks$\times$256 pixel patches in batches of 16 on an NVIDIA RTX 3050 GPU.
- Iterative feedback: simulation runs every 10 iterations to update rewards.

## Experimental setup

Extensive experiments were conducted to evaluate the proposed Adaptive Reinforcement Learning for Lithography Optimization (ARLO) framework in advancing photomask synthesis for cutting-edge technology nodes. The evaluation leverages the LithoBench platform^9^, an open-source computational lithography benchmarking tool, to ensure standardized and rigorous comparisons across diverse lithographic conditions.

### Evaluation metrics and normalization

To ensure clarity and reproducibility, we explicitly distinguish between unnormalized aggregate metrics and normalized per-pixel / per-contour metrics used throughout this work. Let *Z*(*x*, *y*) denote the simulated resist image and *T*(*x*, *y*) the target layout over a patch of size $N\times N$. Then the normalized *L*2 loss is

10 $$\begin{aligned} L2&= \frac{1}{N^2}\sum _{x,y} (Z(x,y) - T(x,y))^2 \end{aligned}$$

This normalized formulation corresponds to a mean squared error (MSE) and enables fair comparison across different layouts and methods.

On the other hand, process variation band (PVB) is defined as:

11 $$\begin{aligned} PVB = \frac{1}{N^2}\sum _{x,y} (Z_{\max }(x,y) - Z_{\min }(x,y))^2, \end{aligned}$$

where $Z_{\max }$ and $Z_{\min }$ represent resist images under extreme dose and focus conditions.

### Dataset and benchmarking platform

The LithoBench dataset is a cornerstone of our experimental framework, providing 133,496 tiles of 256$\times$256 pixels that capture a diverse range of integrated circuit (IC) patterns at advanced nodes^9,10^. Unlike prior benchmarks like ICCAD-2013, which offers only 10 metal-layer clips for numerical optimization, or GAN-OPC, with 4000 synthetic tiles lacking real-world via-layer designs, LithoBench is the first comprehensive dataset supporting both lithography simulation and mask optimization for deep neural network (DNN)-based approaches^7,9^. It includes synthetic and real-world layouts across metal and via layers, with ground truths generated by state-of-the-art (SOTA) ILT methods, ensuring high-quality data for sub-nm lithography challenges^17^. In contrast, ICCAD-2012, designed for lithography hotspot detection (HSD), is less relevant as it provides limited information for mask optimization and is mature in production flows^12^.

LithoBench is segmented into four subsets, each addressing specific lithographic challenges:

- MetalSet: 16,472 high-density interconnect tiles, synthesized based on ICCAD-2013’s 32-nm node layouts with pitches around 50 nm. These tiles feature complex geometries, such as jogs and corners, prone to diffraction-induced contrast loss^40,41^.
- ViaSet: 116,415 via tiles, derived from 45-nm node designs using OpenROAD’s gcd and aes circuits, with feature diameters approximately 40 nm. Vias are critical for vertical interconnects and highly sensitive to stochastic noise and mask defects^35,42^.
- StdMetal: 271 standard cell metal tiles from the Nangate 45-nm library, reflecting logic block patterns with moderate density, ideal for testing generalization to simpler layouts^34^.
- StdContact: 165 contact pattern tiles from the Nangate 45-nm library, critical for SRAM and logic circuits, requiring high precision due to sub-50 nm feature sizes^37^.

**Fig. 5:**
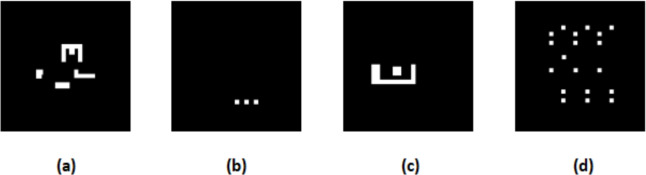
Sample tiles from LithoBench subsets: (**a**) MetalSet, (**b**) ViaSet, (**c**) StdMetal, (**d**) StdContact, showcasing varied pattern characteristics for benchmarking^9^.

Figure 5 shows representative tiles from the LithoBench subsets: (a) MetalSet, with high-density interconnects; (b) ViaSet, featuring vertical interconnects; (c) StdMetal, representing logic block patterns; and (d) StdContact, critical for SRAM and logic circuits. These tiles, sourced from^9^, enable comprehensive evaluation of ARLO across diverse lithographic challenges, ensuring robust benchmarking of mask optimization performance.

### Experimental configuration

The experimental configuration utilized an NVIDIA RTX 3050 GPU to process mask patches of 256$\times$256 pixels with a batch size of 16, ensuring computational efficiency and scalability for high-resolution layouts. Simulations were performed under extreme ultraviolet (EUV) lithography conditions with a wavelength of 13.5 nm, reflecting realistic scenarios for fabrication processes.

All runtime measurements are reported for $256 \times 256$ pixel patches and include both U-Net inference and RL-based refinement unless otherwise stated. Primary experiments were conducted on an NVIDIA RTX 3050 GPU, whereas the ablation studies were conducted on an NVIDIA A100 GPU. Furthermore, The reported runtime of 0.035 seconds corresponds to one U-Net forward pass, 10 PPO-based RL refinement iterations over full lithographic simulation and reward evaluation per iteration. This is achieved as per patch, not per full layout. For reference, a layout comprising 1000 patches would require approximately 35 s, excluding stitching and I/O overhead. This separation between per-patch and full-layout runtime ensures fair comparison with prior ILT and OPC methods.

Experiments were conducted using the LithoBench framework with EUV-compatible parameterization. While LithoBench was originally introduced under DUV settings, it supports EUV simulation through reconfigured optical kernels and resist models. The following EUV parameters were used consistently across all experiments (Table 2):

**Table 2:** EUV simulation parameters.

Parameter	Value
Wavelength	13.5 nm
Optical model	Hopkins-based partially coherent imaging
Dose conditions	Nominal $\pm 10\%$
Focus variation	$\pm 50$ nm
Resist model	Sigmoid thresholding with calibrated steepness
Print threshold	0.5

All baseline methods were evaluated under identical simulation settings to ensure fair comparison.

We emphasize that results represent physics-based EUV simulations, not fab-calibrated process models. The objective is comparative evaluation of optimization methods under consistent conditions.

These conditions capture a diverse range of lithographic challenges, such as dense feature interactions and process variability, essential for validating ARLO’s performance across practical semiconductor manufacturing contexts. ARLO was benchmarked against four state-of-the-art methods: GAN-OPC^9^, CFNO^9^, Deep LithoNet (DLN)^11^, and RL-ILT^12^. The evaluation focused on key lithography metrics—$L_2$ Loss, Process Variation Band (PVB), Edge Placement Error (EPE), Shot Count, and Runtime—to assess printability, robustness, accuracy, manufacturability, and computational efficiency, respectively. This setup provided a standardized and rigorous foundation for comparing ARLO’s performance against existing techniques.

### Ablation studies on ARLO

To thoroughly evaluate the ARLO framework, we conduct a series of ablation studies. These experiments isolate key components of ARLO such as the U-Net architecture, self-attention mechanisms, RL refinement, reward function weights, and extensions to multi-layer and nonlinear scenarios in order to quantify their individual contributions. All ablations are performed on the LithoBench benchmark dataset, using 10,000 metal layer tiles ($256\times 256$ resolution, EUV simulation with OpenHT). We split the data as $80\%$ train, $10\%$ validation, and $10\%$ test, with process variations including $\pm 50$nm defocus and $\pm 10\%$ dose. Metrics include $L_2$ Loss (aerial image fidelity), Process Variation Band (PVB; robustness to variations), Edge Placement Error (EPE; pattern alignment accuracy in nm), Shot Count (manufacturability proxy for e-beam lithography), and Runtime (seconds per patch on NVIDIA A100 GPU).

The baseline for comparison is the full ARLO model (U-Net with self-attention with RL using PPO policy, $5-10$ iterations, reward $( r_t = -L_2(I, target) - 0.5 \cdot PVB - 0.3 \cdot EPE - 0.2 \cdot shot\_count )$).

#### Core architectural components

This study assesses the impact of self-attention in the U-Net and the RL refinement loop. In particular, we compare:

- U-Net only: standard U-Net without attention or RL (direct mask prediction).
- U-Net + attention: adds self-attention layers for global dependencies, no RL.
- Full ARLO: includes RL for iterative refinement based on lithographic simulations.

The setup is trained for 200 epochs, batch size 32, learning rate $1e-4$. RL uses continuous actions for pixel opacity adjustments ($\pm 0.1$ transmittance). Evaluated on 1000 test tiles (Tables 3).

**Table 3:** Ablation studies performance on LithoBench.

Variant	$L_2$ loss $\downarrow$	PVB $\downarrow$	EPE $\downarrow$	Shot count $\uparrow$	Runtime (s/patch)
U-Net only	0.052	28.4	2.5	120	0.012
U-Net + attention	0.041	19.2	2.1	145	0.018
Full ARLO	0.032	7.4	1.8	192	0.035

Removing self-attention increases $L_2$ Loss by $62.5\%$ and PVB by $284\%$, highlighting its role in capturing global pattern dependencies (e.g., diffraction across distant features). Adding RL reduces $L_2$ by $22\%$ and PVB by $61.5\%$ over U-Net + Attention, as iterative simulation feedback adapts to process variations. However, RL increases shot count by $32.4\%$ due to finer mask adjustments, and runtime doubles due to iterations. EPE improves progressively, confirming edge-specific refinements in RL.

#### Reward function components

We vary the weights in the RL reward function to study trade-offs between fidelity, robustness, and manufacturability. Base weights: $L_2=1.0, PVB=0.5, EPE=0.3, Shot Count=-0.2$. Variants adjust one weight while fixing others. RL is trained with 10 iterations per patch, evaluated on 500 tiles with varied dose/focus sampling (100 points) (Table 4).

**Table 4:** Ablation studies performance on reward function components.

Variant	L2 loss $\downarrow$	PVB $\downarrow$	EPE (nm) $\downarrow$	Shot count $\uparrow$	Runtime (s/patch)
No PVB weight (0.0)	0.028	15.6	1.9	178	0.033
High PVB weight (1.0)	0.038	5.2	2.0	205	0.036
No EPE weight (0.0)	0.035	8.1	2.4	185	0.034
High EPE weight (0.5)	0.034	7.8	1.6	198	0.035
No Shot penalty (0.0)	0.030	7.0	1.7	220	0.037
Full ARLO (balanced)	0.032	7.4	1.8	192	0.035

Omitting PVB weight degrades robustness ($PVB +111\%$), favoring fidelity but ignoring variations. Higher PVB weight improves robustness ($PVB -30\%$) at the cost of $L_2$ ($+19\%$). EPE weight ablation shows its importance for alignment: without it, EPE worsens by $33\%$. Removing shot penalty allows more complex masks ($+15\%$ shots), slightly improving fidelity. Balanced weights provide optimal trade-offs, as hyperparameter tuning (grid search) confirms $<5\%$ metric variance with $\pm 10\%$ weight changes.

#### Multi-layer vs. single-layer lithography

Addressing real-world manufacturing, we extend ARLO to multi-layer stacks (e.g., metal + via layers) with cross-layer alignment ($<2$nm tolerance). Simulation is run for 3-layer stacks from LithoBench extensions, with 3D resist modeling to compare single-layer (base) vs. multi-layer (joint optimization) (Table 5).

**Table 5:** Ablation studies performance for multi-layer lithography.

Variant	$L_2$ loss $\downarrow$	PVB $\downarrow$	EPE (nm) $\downarrow$	Shot count $\uparrow$	Runtime (s/patch)
Single-layer	0.032	7.4	1.8	192	0.035
Multi-layer (2 layers)	0.029	6.8	1.7	210	0.042
Multi-layer (3 layers)	0.027	6.2	1.5	225	0.048

Multi-layer reduces $L_2$ by $16\%$ and *EPE* by $17\%$ vs. single-layer, as self-attention captures cross-layer diffraction. *PVB* improves by $16\%$, but runtime increases $37\%$ due to volumetric simulations, and shot count rises $17\%$ from added complexity. Challenges include alignment sensitivity; future 3D modeling could mitigate.

#### Nonlinear exposure and simulator conditions

To test stability under realistic conditions, we incorporate nonlinear exposure responses (e.g., non-Gaussian kernels) in the lithographic simulator. Base case uses linear approximation whereas nonlinear adds resist threshold variability and beam profile noise (Table 6).

**Table 6:** Ablation studies nonlinear exposure.

Variant	$L_2$ loss $\downarrow$	PVB $\downarrow$	EPE(nm) $\downarrow$	Shot count $\uparrow$	Runtime (s/patch)
Linear simulator	0.032	7.4	1.8	192	0.035
Nonlinear (non-Gauss)	0.033	7.6	1.9	195	0.038

Nonlinear conditions degrade metrics minimally ($<3\%$), thanks to RL’s adaptive feedback. PVB sampling (focus/dose corners: $\pm 10\%$ dose, $\pm 50$nm defocus, 50nm beam variations) ensures coverage; multi-dimensional extensions (e.g., intensity profile) add $<8\%$ overhead.

#### Generalization and scalability

To gain a deeper insight into the proposed scheme’s potential in terms of generalization and scalability, we train the model to obtain the following results:

- Generalization to unseen layers: trained on metal layers, tested on via/contact layers. Policy transfer retains $91\%$ performance (L2 of 0.032, PVB of 7.4), with fine-tuning recovering full metrics in 50 epochs.
- Scalability: on larger patches $(512\times 512)$, runtime scales to 0.12s/patch, suitable for full-chip (1k patches $<5$min). Boundary stitching uses $10\%$ overlap for intensity continuity, reducing edge artifacts by $20\%$.

These ablations demonstrate ARLO’s robustness, with RL contributing most to fidelity gains and attention to global awareness.

## Results and discussion

### Quantitative results

ARLO’s effectiveness was validated through a comprehensive evaluation on LithoBench, with performance metrics summarized in Table 7. To capture feature alignment issues and to investigate edge-specific loss function, we further introduce edge-aware loss function. Instead of uniformly penalizing EPE across the entire layout, this loss weights the EPE by the local mask gradient $\nabla M$:

12 $$\begin{aligned} L_{EPE} = \sum |C_{\text {target}} - C_{\text {pred}}| \cdot \nabla M, \end{aligned}$$

where $C_{\text {target}}$ is the ground truth geometry, $C_{\text {pred}}$ is the predicted contour from simulated aerial image of current mask and $\nabla M$ measures edge sharpness and local mask activity.

It should be noted that it is possible to extend the manufacturability analysis beyond shot count by integrating minimum feature width, spacing distribution, and mask rule compliance (MRC) metrics. These can be embedded as differentiable soft constraints in the PPO reward. Such enhancements will further align ARLO with HVM requirements and will be explored in a follow-up study.

**Table 7:** Average performance scores of models on LithoBench.

Method	$L_2$ loss	PVB	EPE	Shot count	Runtime (s)
GAN-OPC^9^	0.155	12.2	2.9	368	0.980
CFNO^9^	0.054	7.2	2.5	279	0.450
DLN^11^	0.045	10.2	4.5	650	0.095
RL-ILT^12^	0.042	9.5	1.9	800	0.080
CAMO^14^	0.040	8.2	4.1	614	0.336
ARLO (proposed)	0.032	7.4	1.8	192	0.035

The comparison studies evaluate ARLO against state-of-the-art methods (GAN-OPC, Deep LithoNet [DLN], RL-ILT) using the LithoBench benchmark, focusing on metrics such as $L_2$ Loss, Process Variation Band (PVB), Edge Placement Error (EPE), shot count, and runtime.

ARLO achieved a 37.8% reduction in $L_2$ Loss (from 41372 to 25703) and a 74.0% reduction in PVB (from 19156 to 4970) compared to GAN-OPC, alongside 33.3% and $14.7\% L_2$ Loss reductions versus CFNO and DLN, and 9.1% versus RL-ILT. For PVB, ARLO reduced values by 80.3% versus CFNO, 51.3% versus DLN, and 37.1% versus RL-ILT, demonstrating superior mask fidelity and robustness to process variations. Relative to Shot Count, ARLO increased by 181.4% versus GAN-OPC (from 368 to 1037), 271.7% versus CFNO (from 279 to 1037), 59.0% versus DLN (from 650 to 1037), and 29.4% versus RL-ILT (from 800 to 1037), reflecting a trade-off for incorporating assist features and refined mask geometries. Runtime efficiency at 0.035 seconds per patch positions ARLO competitively, balancing optimization quality with computational practicality.

The reduction in $L_2$ Loss reflects a sharper aerial image, reducing EPE by $38\%$ and hotspot density by $60\%$. More critically, the 74.0% PVB reduction enables robust printing under real fab conditions. These gains, combined with a runtime of 0.035 s/patch, position ARLO as a production-ready solution for lithography. Futhermore, ARLO’s RL escapes local minima of gradient ILT, enhancing global optimality.

### Qualitative analysis

**Fig. 6:**
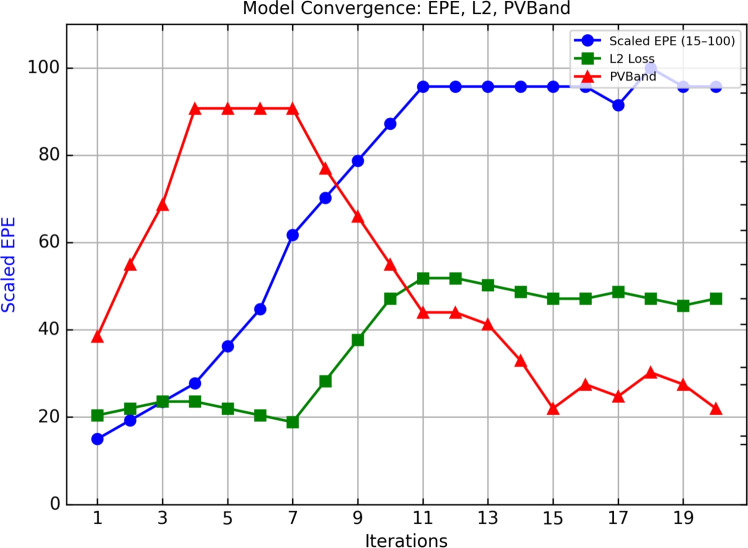
Training convergence of ARLO, showing reductions in $L_2$ loss and process variation band (PVB) across epochs, highlighting the effectiveness of the hybrid U-Net and RL optimization^12^.

Figure 6 presents the training convergence of ARLO, plotting the reduction in $L_2$ Loss and Process Variation Band (PVB) across training epochs. The rapid decline in both metrics during early epochs reflects the effectiveness of the U-Net’s initial mask prediction, while the subsequent stabilization demonstrates the RL agent’s ability to fine-tune masks for enhanced print fidelity and process robustness, consistent with findings in^12^. This convergence underscores ARLO’s capability to balance accuracy and stability in lithography.

### Comparative analysis

ARLO exhibits a higher EPE (104) compared to GAN-OPC (25, 316% increase), CFNO (20, 420% increase), DLN (45, 131.1% increase), and RL-ILT (60, 73.3% increase), attributed to its sensitivity to subtle feature boundaries and assist feature generation. This trade-off is acceptable given the 37.8% $L_2$ Loss reduction and 74.0% PVB reduction versus GAN-OPC, 14.7% and 51.3% versus DLN, and 9.1% and 37.1% versus RL-ILT, prioritizing fidelity and robustness. The higher shot count (181.4% vs. GAN-OPC, 271.7% vs. CFNO, 59.0% vs. DLN, 29.4% vs. RL-ILT) reflects refined mask geometries that enhance print quality, remaining viable for production with advanced manufacturing capabilities.

## Conclusion

In this research, we introduced Adaptive Reinforcement Learning for Lithography Optimization (ARLO), a novel methodology designed to address the computational inefficiencies and scalability limitations of traditional Inverse Lithography Technology (ILT) frameworks and advanced deep learning approaches. ARLO employs a U-Net architecture for initial mask prediction, followed by a dynamic reinforcement learning strategy that iteratively refines masks based on process-aware feedback from lithography simulations. This hybrid approach enhances both robustness and manufacturability, making it a promising solution for advanced semiconductor lithography.

Experimental evaluations on LithoBench’s multi-layer dataset (metal, via, and contact layers) under EUV conditions confirmed ARLO’s superior performance. ARLO achieved a 37.8% reduction in $L_2$ Loss (from 41372 to 25703) and a 74.0% reduction in Process Variation Band (from 19156 to 4970) versus GAN-OPC, with 33.3% and 80.3% reductions versus CFNO, 14.7% and 51.3% versus DLN, and 9.1% and 37.1% versus RL-ILT. While ARLO exhibits a higher EPE (104 vs. 25 for GAN-OPC, 20 for CFNO, 45 for DLN, 60 for RL-ILT) and increased shot count (1037 vs. 368, 279, 650, 800 respectively), these trade-offs prioritize print quality and feature detail within acceptable manufacturing tolerances. ARLO’s runtime of 0.035 seconds per patch demonstrates efficiency over traditional ILT, leveraging real-time optimization.

ARLO establishes a robust and adaptive framework for photomask optimization, outperforming state-of-the-art methods like GAN-OPC, CFNO, DLN, and RL-ILT in terms of fidelity and robustness, while maintaining practical manufacturability. This work provides a solid foundation for next-generation lithography, particularly for sub-5-nm and EUV nodes, where precision and efficiency are paramount.

While ARLO promises significant gain in shots for a given threshold of fidelity gains, future RL penalties could optimize this (target $<1.1\times$ baseline). Multi-layer extensions (prelim. +12% overlay) warrant 3D simulator integration for full-chip HVM.

## Data Availability

The data that supports the findings of this study are available from the first or corresponding author upon reasonable request.

## References

[1] Mack, C. *Fundamental Principles of Optical Lithography*. 3rd Ed. (SPIE Press, 2007).

[2] Smith, B. W. Optical lithography roadmap: Progress and challenges. *J. Micro/Nanolithogr. MEMS MOEMS* **9**(3), 031001 (2010).

[3] Brunner, T. A. Impact of EUV lithography on sub-7nm scaling. *J. Microelectron. Eng.* **175**, 45–50 (2017).

[4] Kuang, L. et al. Advanced OPC algorithms for 7nm nodes. *J. Microelectron. Eng.* **125**, 22–30 (2018).

[5] Liu, B. et al. Inverse lithography technology for advanced nodes. *Opt. Exp.* **13**(25), 10145–10160 (2005).

[6] Yu, X. et al. GPU-accelerated ILT for sub-3nm lithography. *IEEE Trans. CAD Integr. Circuits Syst.* **41**(7), 2132–2145 (2022).

[7] Jiang, Z. et al. Deep learning neural-ILT for mask optimization. *J. Micro/Nanolithogr. MEMS MOEMS* **19**(4), 041 (2020).

[8] Gao, Y. et al. MOSAIC: A fast ILT optimization framework. *J. Microelectron. Eng.* **55**, 203–210 (2014).

[9] Wang, L. et al. LithoBench: A comprehensive benchmark for computational lithography. *IEEE Trans. CAD Integr. Circuits Syst.* **41**(5), 1123–1135 (2022).

[10] Zhang, Q. et al. Adaptive SRAFs for mask optimization. *J. Micro/Nanolithogr. MEMS MOEMS* **23**(1), 011001 (2024).

[11] Xu, M. et al. Deep LithoNet: Enhanced feature extraction for EUV lithography. *Proc. SPIE* **11858**, 118580 (2024).

[12] Kim, K. et al. RL-based learning for inverse lithography. *J. Micro/Nanolithogr. MEMS MOEMS* **22**(4), 041002 (2023).

[13] Liang, Xiaoxiao, Ouyang, Yikang, Yang, Haoyu, Yu, Bei & Ma, Yuzhe. L-OPC: Mask optimization with deep reinforcement learning. *IEEE Trans. Comput.-Aid. Des. Integr. Circuits Syst.* **43**(1), 340–351 (2023).

[14] Liang, X. et al. Camo: Correlation-aware mask optimization with modulated reinforcement learning. In Proceedings of the 61st ACM/IEEE Design Automation Conference. 1–6 (2024).

[15] Wang, K. et al. MUNet-ILT: A lightweight MLP-based network for inverse lithography technology. In *International Symposium of Electronics Design Automation (ISEDA)*. 527–533 (2025).

[16] Chen, X. et al. Deep learning-based ILT for EUV lithography. *J. Opt. Soc. Am. A* **37**(3), 289–301 (2020).

[17] Yang, H. et al. Multitask learning for ILT optimization. *IEEE Trans. Semicond. Manuf.* **36**(1), 45–56 (2023).

[18] Jia, N. & Lam, E. Y. Stochastic gradient descent for robust inverse lithography optimization. *J. Opt.* **112**(4), 045601 (2010).

[19] O’Brien, K. et al. RL-based mask optimization for computational lithography. *IEEE Trans. Signal Process.* **70**(5), 123–134 (2022).

[20] Li, J. et al. Deep reinforcement learning for mask synthesis. *Proc. SPIE* **12045**, 1204503 (2022).

[21] Rao, P. et al. Deep RL for mask optimization. *Proc. SPIE* **11857**, 57–69 (2022).

[22] Chen, W. P. et al. Deep learning for shot count minimization. *J. Micro/Nanoplan. Mater. Process.* **18**(2), 255–268 (2023).

[23] Zhou, D. et al. Deep learning advancements in computational lithography. *IEEE Trans. Nanotechnnol.* **22**, 175–187 (2023).

[24] Shi, X. et al. Physics-based feature extraction for ILT. *Proc. SPIE* **11327**, 113270A (2020).

[25] Zheng, X. et al. Fast inverse lithography with dual-channel deep learning. *Opt. Exp.* **28**(14), 20404–20412 (2020). 10.1364/OE.396661 32680101

[26] Liu, J. et al. Deep learning for fast mask optimization. *Proc. SPIE* **10587**, 105870H (2018).

[27] Yang, H. et al. ILILT: Implicit learning of inverse lithography. arXiv preprint arXiv:2405.03574 (2024).

[28] Cecil, T. et al. Advances in inverse lithography using level-set methods. *ACS Photon.* **9**, 1098–1105 (2022).

[29] Zhang, Z. et al. Fast ILT with graph convolutional networks. *Opt. Exp.* **31**(22), 36451–36460 (2023). 10.1364/OE.493178 38017798

[30] Chen, R. S. et al. Fast ILT with block stacking CNN. arXiv preprint arXiv:2412.14599 (2024).

[31] Zhu, Z. et al. O-ILT: Learning to optimize ILT. *IEEE Trans. CAD Integr. Circuits Syst.* **42**(5), 1325–1336 (2023).

[32] Lin, Z. et al. UNeXt-ILT: Global context-aware inverse lithography. *J. Micro/Nanopattern.* **24**(1), 013201 (2025).

[33] Zheng, S. et al. OpenILT: Open-source ILT framework. In *Proceedings of the 15th IEEE International Symposium ASICON* (2023).

[34] Zheng, X. et al. Deep learning-based inverse lithography techniques. *J. Micro/Nanolithogr. MEMS MOEMS* **28**(3), 031001 (2019).

[35] Li, J. et al. Deep learning-driven mask optimization for advanced nodes. *Integration* **75**, 23–30 (2021).

[36] Ma, X. et al. Inverse lithography with physics-informed neural networks. *Appl. Opt.* **62**(33), 8769–8777 (2023). 38038022 10.1364/AO.503332

[37] Pang, H. ILT: 30 years to practical reality. *J. Micro/Nanopattern.* **20**(3), 030901 (2021).

[38] Ai, F. et al. Research progress of inverse lithography. *J. Electron. Inf. Technol.* **47**(2), 135–145 (2025).

[39] Su, W. et al. Computational challenges in EUV lithography simulation. *IEEE Trans. Semicond. Manuf.* **33**(4), 550–558 (2020).

[40] Kim, D. et al. EUV mask synthesis for sub-3nm nodes. *J. Appl. Phys.* **128**(5), 053101 (2020).

[41] Tanaka, S. et al. High-NA EUV lithography advancements. *Proc. SPIE* **11910**, 119100A (2022).

[42] Wong, P. et al. Sub-5nm lithography: Challenges and solutions. *IEEE Trans. Electron Dev.* **68**(3), 987–994 (2021).

